# Relationship between humoral response against hepatitis C virus and disease overcome

**DOI:** 10.1186/2193-1801-3-56

**Published:** 2014-01-27

**Authors:** Carine Brakha, Philippe Arvers, Florent Villiers, Alice Marlu, Arnaud Buhot, Thierry Livache, Roberto Calemczuk, Jean-Pierre Zarski, Christian L Villiers, Patrice N Marche, Marie-Bernadette Villiers

**Affiliations:** INSERM, U823, Institut A. Bonniot, BP 170 Cedex 9, F-38042 Grenoble, France; HIA Desgenettes, 108 Bd Pinel Cedex 03, Lyon, F-69275 France; Department of Cell Biology and Molecular Genetics, University of Maryland, College Park, MD 20742 USA; Pôle Digidune, Centre Hospitalier Universitaire de Grenoble, La Tronche, F-38700 France; INAC, SPrAM (UMR 5819, CEA, CNRS, UJF), INAC/CEA Cedex 09, Grenoble, F-38054 France; Université J. Fourier, UMR-823, F-38042 Grenoble, France

**Keywords:** Antibody, Epitope, Hepatitis C virus, Peptide chip, SPR imaging

## Abstract

**Abstract:**

Hepatitis C virus infection leads to liver disease whose severity can range from mild to serious lifelong illness. However the parameters involved in the evolution of the disease are still unknown. Among other factors, the virus-elicited antibody profile is suspected to play a role in the outcome of the disease. Analysis of the relationship between anti-virus antibodies and disease state requires the analysis of a large number of serums from patients (hepatitis C virus+) and of epitopes from the viral proteins. Such a study would benefit from microarray-based screening systems that are appropriate for high-throughput assays.

We used a method combining peptide chips and surface plasmon resonance imaging previously shown to be suitable for analyzing complex mediums and detecting peptide-protein interactions. 56 peptides covering the entire viral proteome were grafted on chips and their interaction with antibodies present in the 68 injected serums from infected and non-infected donors was measured. Statistical analyses were conducted to determine a possible relationship between antibodies (specificity and amount) and disease states.

A good discrimination between infected and non-infected donors validated our approach, and several correlations between antibodies profiles and clinical parameters have been identified. In particular, we demonstrated that ratios between particular antibodies levels allow for accurate discrimination of patients according to their pathologic states.

**Conclusion:**

Humoral response against hepatitis C virus linear epitopes is partly modified according to the disease state. This study highlights the importance of considering relative quantities of antibodies with different specificities rather than the amount of each antibody.

**Electronic supplementary material:**

The online version of this article (doi: 10.1186/2193-1801-3-56) contains supplementary material, which is available to authorized users.

## Introduction

Although hepatitis C virus (HCV) induces a strong humoral response within a few weeks after infection, only 25-30% of the infected individuals resolve the disease whereas the rest develop chronic hepatitis C. Among the latter group, 20% progress to cirrhosis, whereof 5% further evolve towards hepatocellularcarcinoma (HCC) (Zein [2003]). The parameters determining the outcome of the infection remain unknown. Indeed, cellular and humoral immune responses work together to protect individual against infections, but are also suspected to induce liver damage associated with HCV infection (reviews (Gremion and Cerny [2005]; Irshad et al. [2008])). As antibodies (Ab) play a key role in immune response, they are believed to be involved in the outcome of HCV disease. It is well known that HCV elicits antibodies against both structural and non-structural proteins (Gabrielli et al. [1996]; Gutierrez et al. [2011]; Sillanpaa et al. [2009]) and several studies have investigated a possible relationship between immuno-reactivity and clinical features. While a correlation between viremia and Ab titres has been established (Bassett et al. [1998]; Lohr et al. [1996]; Yuki et al. [1994]), studies attempting to discriminate between acute and chronic phases or between responders and non-responders to treatment led to more controversial results (Araujo et al. [2011]; Hilfenhaus et al. [1992]; Lohr et al. [1996]). However, most of these studies have focused on the occurrence of Ab directed against different viral proteins without regards to their quantification. Moreover, they are often limited by the use of recombinant large proteins or polypeptides as probes to analyse anti-HCV Ab, which allows for a global, rather than precise determination of their specificity. A more accurate analysis using smaller peptides as probes could increase the number of identified epitopes targeted by anti-HCV Ab while also indicating their specificity. Although allowing for the detection of linear epitopes only, this approach can be useful in the case of HCV infection since human Ab are mainly directed against linear epitopes of the virus (Sallberg et al. [1992]). It also benefits from the recent development of microarray-based screening systems. This technology has found its first applications in gene expression screening, and DNA/RNA microarrays have been used in clinical research for predicting the outcome of the disease (van’t Veer et al. [2003]; Zhao et al. [2006]), response to treatment (Augustine et al. [2009]) or for determining cancer class (Golub et al. [1999]). More difficult to implement, protein microarrays have emerged later and still remain rarely used: the complexity and heterogeneity of the probes together with the necessity to preserve their conformational folding impair their benefit. Peptide microarrays are therefore a good alternative to protein arrays and are suitable for large screening. They have demonstrated their reliability in detecting specific Ab content in serums, allowing one to test both a wide range of potential epitopes and a large number of samples (Cherif et al. [2006]; Maksimov et al. [2012]; Perez-Gordo et al. [2012]; Villiers et al. [2010]; Villiers et al. [2011]).

Using such a technology with a set of probes comprising 56 peptides spanning the entire HCV proteome, our aim was to analyse the HCV-elicited immune response in 45 HCV-positive donors. Both the occurrence and magnitude of each Ab response were determined using surface plasmon resonance imaging (SPRi) to detect Ab-peptide interactions. We investigated a possible relationship between immuno-reactivity and various clinical parameters such as state of the disease (chronic, cirrhotic, HCC), viremia, ALAT (alanine amino-transferase) level, and Metavir scores. A good discrimination between HCV-positive and negative donors validated our approach, and several correlations between Ab profiles and clinical parameters have been identified. In particular, we demonstrated that ratios between particular Ab levels allow for accurate separation of the three states of the disease.

## Materials and methods

### Serums

Human serums were provided by the Etablissement Français du Sang (Grenoble, France) and the Centre Hospitalo-Universitaire, Grenoble, France. The cohort included 15 serums from healthy donors, 8 serums from HCV-negative patients (HCV-) with alcoholic cirrhosis (HCC-OH) and 45 serums from HCV-positive patients (HCV+) infected with genotype 1b, at various stages of the disease: chronic hepatitis C (15 serums), cirrhosis (16 serums) and hepatocellularcarcinoma (HCC) (14 serums). Clinical data are shown in Additional file 1. All participants provided written informed consent. Rabbit immune serum against HCV peptide C131 (control serum) was prepared by NeoMPS (Strasbourg, France).

### Peptides

Peptides were synthesized by Altergen (Bischheim, France) with a pyrrole-modified –NH_2_ (Villiers et al. [2009]). Fifty-six peptides are derived from HCV genotype 1b (ExPASy accession number P26663), one from hen egg lysozyme (P00698, control peptide).

### SPR analysis

Glass prisms coated with a 50 nm gold layer were obtained from HORIBA-Scientific (Palaiseau, France). Peptides (100 μM) were grafted in duplicate on the biochip surface by electrochemical copolymerization (Villiers et al. [2011]), using an Omnigrid Micro-robotic arrayer (Horiba-Scientific). Ab binding on the grafted peptides was detected using Surface Plasmon Resonance imaging (SPRi). This optical technique allows for the detection of protein-peptide interaction through a change in the intensity of the reflected light. Successive serum injections (1:50 in PBS) were performed as previously described (Villiers et al. [2011]). Stabilization of the peptide chip was ensured by twelve blank injection/regeneration cycles before sample analysis. Periodic injections of control rabbit anti-C131 serum (1:200) allowed to monitor the reduction of signal along successive injections and to model it using polynomial curve. This curve was used to determine a correction factor to be applied to sample injections (Villiers et al. [2011]). SPR signals were monitored at 810 nm using a surface plasmon resonance imager SPRi-Plex from Horiba-Scientific. Measurements were performed using SPRi-dedicated software (Horiba-Scientific).

### Statistical analysis

Ratio calculations and Mann–Whitney tests were performed using R v2.15.1. Hierarchical clustering (Pearson’s correlation, average linkage) and heatmaps were generated using Genesis software (release 1.7.6, Graz University of Technology, Austria) (Sturn et al. [2002]).

Multivariate data analysis was conducted using Soft Independent Modeling of Class Analogy (SIMCA) with Partial Least Square (PLS) regression and Discriminant Analysis (DA): PLS-DA is a regression analysis that takes advantage of class information to attempt to maximise the separation between groups of observations (SIMCA-P, Umetrics AB). R2 is an estimate of the proportion of variance explained by the model and Q2 an estimate of the predictive ability of the model.

## Results

### Selection of the grafted peptides

To analyse the specificity of anti-HCV Ab in serums, peptides to be used as probes were selected according to various criteria. Sequences were derived from genotype 1b, the most common in Europe (Chen and Weck [2002]). The length of the peptides was about 20 residues to ensure good accessibility. Furthermore, assuming that potential antigenic regions are predominantly located at the surface of the proteins (Chen et al. [2007]; Novotny et al. [1986]), hydrophilic peptides were favoured. The high rate of mutation occurring in HCV affects epitope recognition by the Ab. Thus, peptide sequences were chosen in regions exhibiting relatively conserved sequences across genotypes. In order to have an overview of the humoral response against conserved linear epitopes, the selected peptides (Additional file 2) were derived from all HCV proteins.

### Overall analysis of antibody-targeted epitopes

**Figure 1:**
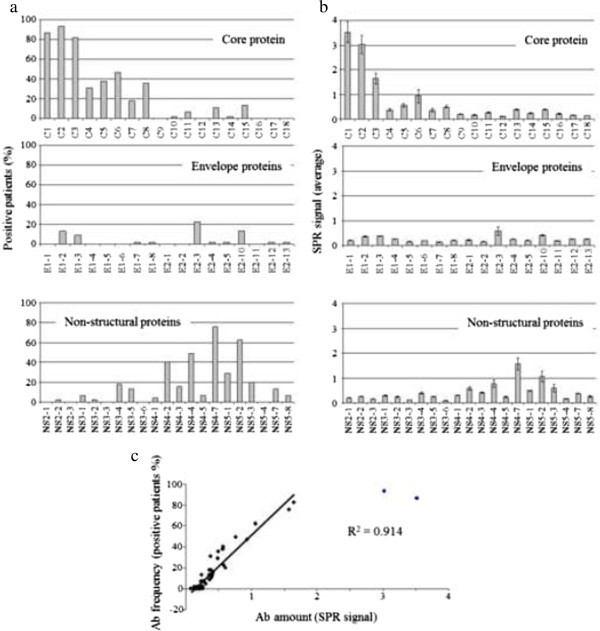
**Targeted epitopes.** 45 HCV + serums were injected on the chip, and the SPR signals obtained from the different grafted peptides were measured. Data represent mean of three independent experiments. **(a)** Percentage of patients with significant SPR signal (≥ 0.5) for each peptide. **(b)** Mean value of SPR signals (change in reflectivity = ΔR) obtained on each peptide for all patients. **(c)** Correlation between Ab amount against a peptide and the percentage of patients with Ab recognizing this peptide

We first analysed the linear epitopes recognized by the Ab present in HCV + serums. As previously described, the SPR signal in the range of 0.5% to 10% detected from each grafted peptide is proportional to the concentration of its corresponding specific Ab present in the sample (Cherif et al. [2006]). Two parameters were analysed for each peptide: the percentage of patients exhibiting SPR signal > 0.5 (i.e. medium/large response (Figure 1a) and the mean value of this signal (Figure 1b). Anti-HCV response is mainly directed against the Core protein, particularly against the first N-terminal aa residues (1–80): more than 80% of the patients possess Ab directed against the first 40 aa. NS4 and NS5A are also well recognized by Ab, though to a lesser extent. The tested peptides from both envelope (E1 and E2) and non-structural NS2, NS3 and NS5B proteins are poorly immunogenic as the signals obtained for these sequences are low and rare. In most cases, the C-terminal portion of the proteins does not elicit a strong Ab response: less than 5% of the patients possess a medium/large amount of Ab against peptides from Core 150–189, E1 271–380, E2 605–702, and NS2 1002–1023. Overall, a good correlation (R^2^ 0.914) is found between Ab frequency and amount (Figure 1c). For the following analyses and to minimize the individual variations in the ability to synthesize Ab, we used normalized data. For each serum, SPR signals were expressed as a percentage of the response obtained for all the peptides tested.

### Analysis of the correlation between the epitopes targeted by Ab

**Figure 2:**
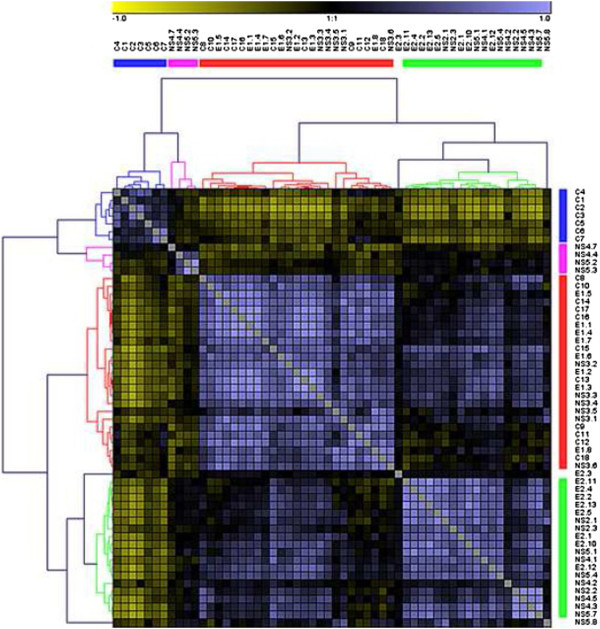
**Correlation between epitopes recognized by the Ab present in tested serums.** Heatmap generated from Pearson’s correlation analysis. Identification of four peptide groups (1–4). Data represent mean of three independent experiments.

First, we evaluated the correlation between the different SPR signals (i.e. Ab amount in each HCV + serum) obtained for each peptide. Hierarchical clustering analysis using the Pearson’s correlation led to the identification of four groups of peptides (Figure 2):

–The first group (blue) includes the first N-Ter peptides from Core (C1-C7).
–The second group (red) includes the remaining peptides from Core (C8-C18), the peptides from E1 and NS3.
–The third group (green) consists of peptides from E2 (except E2-3) and some of those from NS4 and NS5, including the C-Ter NS5-4 and NS5-7.
–The fourth group (pink) is constituted by the remaining peptides from NS4 and NS5 proteins.

These results are confirmed by principal component analysis (data not shown). In this case, NS5-8 is included in group 3, whereas C15 and E2-3 does not fit in any group.

### Correlation between Ab specificity/amount and disease state

**Figure 3:**
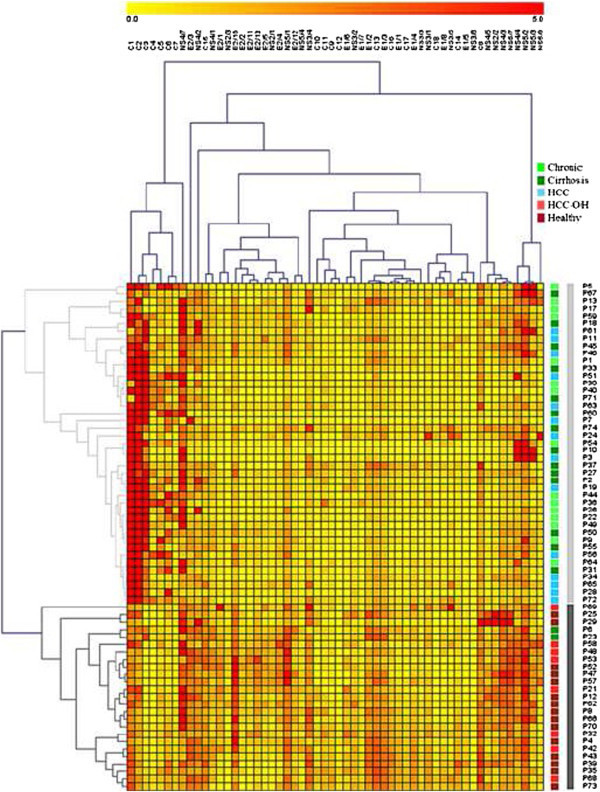
**Peptide probe/serum pairwise SPRI intensity values.** SPRI signal intensities from each peptide probe (columns) were collected after sequential exposure of the chip to various serums (rows) and are displayed as a heatmap. Dendrograms show the hierarchical clustering of the considered variables using the Pearson correlation coefficient, average linkage clustering method. Data correspond to the mean of three independent experiments.

To establish a correlation between the Ab profile of the tested serums and the clinical diagnosis of the corresponding donors, the patients were clustered based on the signal intensity that their serum triggered for all of the 56 peptides. While hierarchical clustering (Pearson’s correlation) successfully discriminates between HCV + and HCV- patients (Figure 3), no clear separation of the different disease states can be established by this approach.

**Figure 4:**
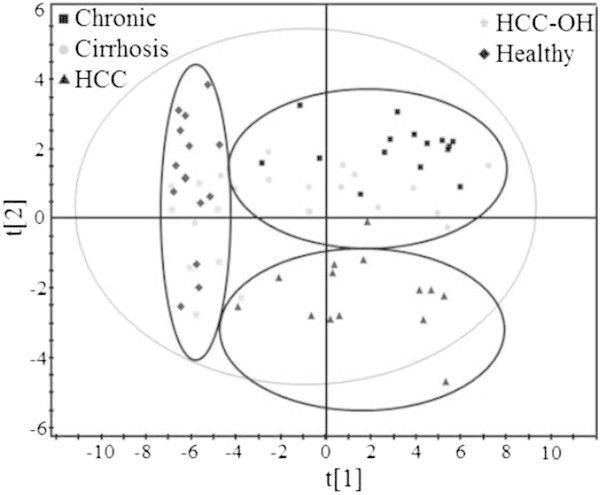
**Correlation between Ab specificity and disease state using partial least square discriminant analysis.** The graph provides the plan which separates the 5 given classes as well as possible, according to the descriptive variables. Data represent mean of three independent experiments.

Thus, we used Partial Least Square Discriminant Analysis to find a model that separates classes of observations (here 5 classes) based on their descriptive variables (peptides). This approach allows us to identify 3 clusters as shown in Figure 4. As expected, a very good separation between HCV- (HCC-OH and healthy) and HCV + serums is observed, with high degree of fit (R^2^ 0.815) and predictability (Q^2^ 0.692). In addition, HCC patients can be grouped into one cluster, leaving chronic and cirrhosis patients together in a third cluster (R^2^ 0.463, Q^2^ 0.215).

Then, we investigated whether it was possible to establish a model for accurate discrimination between all the various states of HCV disease. The entire dataset is divided into a training set (to build a model), and a test set (to validate the model).

Table 1 summarizes 10 different clustering models that were evaluated using this approach. By breaking down the dataset to only two disease states (HCV + and HCV-; Model #1), we were able to obtain more than 95% correct classification, confirming the previously evidenced clusters. ALAT-based (threshold = 2 N; Model #9) and viral load-based (threshold = 600,000 UI; Model #10) clustering were also found to be particularly relevant with a minimum correct classification of 90.9% and 100%, respectively. However regardless of the clustering of the training dataset with respect to the pathology (Models #2 to 5), or with respect to the Metavir scores (Models #6, 7 and 8), no models yielded an accurate classification of the test set.

**Table 1:** **Classification of Ab specificity versus disease state**

Parameter	Model #	Disease state	Number of serums	Correct classification (%)
**HCV**	1	**+**	45	100
		**-**	23	95.7
**Pathology**	2	Chronic hepatitis	15	93.3
		Cirrhosis	16	75
		HCC	14	100
		HCC-OH	8	37.5
		Healthy	15	80
	3	Chronic hepatitis	15	60
		Cirrhosis	16	56.2
		HCC	14	0
		Healthy + HCC-OH	23	100
	4	Chronic hepatitis + Cirrhosis	31	87.1
		HCC	14	0
		Healthy + HCC-OH	23	100
	5	Chronic hepatitis	15	0
		Cirrhosis + HCC	30	90
		Healthy + HCC-OH	23	95.6
**Metavir F (score)**	6	0+1	13	92.3
		2	14	0
		3+4	27	86.4
**Metavir A (score)**	7	1	14	100
		2	17	82.3
		3	7	0
	8	1+2	31	78.6
		3	7	91.7
**ALAT/Norm ALAT**	9	≤ 2N	23	90.9
		> 2N	18	97.7
**Viral load (UI)**	10	< 600 000	8	100
		> 600 000	30	100

### Separation of each disease state from the others

**Figure 5:**
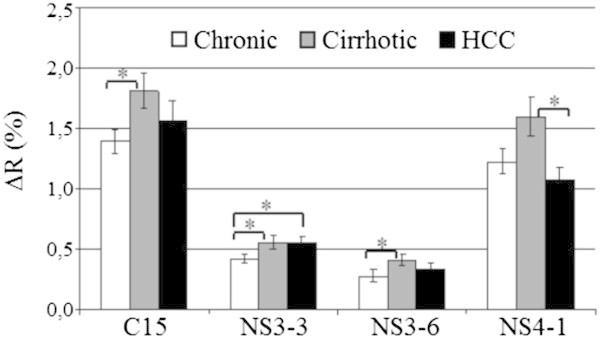
**Peptides differently targeted by Ab with respect to the disease state.** 45 HCV + serums from patients at various stages of the disease were injected on the chip, and SPR signals obtained from the different grafted peptides were measured (change in reflectivity = ΔR). Asterisks denote significantly different signals (pval ≤ 0.05) between groups as evaluated by Mann–Whitney test.

Since the global Ab profile of each patient, as analyzed by PLS-DA, does not allow for valid predictive model of the disease state, we investigated the possibility of using particular Ab as specific markers of the various diagnoses. For each of the peptides, we performed a Mann–Whitney test between the 3 disease groups to evaluate the (dis)similarity of the Ab signal they mediated and to identify Ab which amount can be correlated to a specific state of the disease (healthy patients were not included). Four peptides exhibit significant differences in the signal they triggered between at least 2 patient groups (p < 0.05), whereof only one (NS3-3) can efficiently separate one disease state from the 2 others (Figure 5). Three peptides are significantly more recognized by Ab present in serums from cirrhotic patients than from chronic (C15 and NS3-6) or HCC patients (NS4-1), and Ab recognizing NS3-3 are significantly lower in the case of chronicity compared to both cirrhosis (p 0.048) and HCC (p 0.047).

### Correlation between Ab specificity/amount and various clinical parameters

Humoral response upon HCV infection was analysed using PLS-DA in correlation with various parameters used by clinicians to assess the severity of the disease: Metavir scores, ALAT activity, and viral load. When considering the total amount of all anti-HCV antibodies, regardless of their specificity, no correlation is observed with either ALAT level or viral load (data not shown). However, when we consider each Ab individually, good correlations are observed with ALAT level (threshold 2 N) and with viral load (threshold 600 000 UI), allowing for predictive models leading to more than 90%, and even to 100% correct classification for ALAT and viral load, respectively (Table 1).

### Analysis of SPR signal ratios

To enhance the sensitivity of the study, we decided to analyse the ratio of SPR signals from pairs of peptides rather than from individual peptides. This strategy identifies, among all possible combinations, several Ab ratios for which there is a significant difference (p < 0.05) between one state of the disease and the two others (Table 2 and Additional file 3). Two ratios allow for the separation of cirrhotic patients from chronic and HCC ones, whereas 15 and 14 ratios distinguish chronic and HCC, respectively, from the two other states. Remarkably, one ratio (C11/NS3-3) is shown to separate each state from the others.

**Table 2:** **Ab ratios allowing for a separation of each disease state from the two others (Mann–Whitney analysis)**

Ab ratio^1^	Chronic hepatitis		Cirrhosis		HCC	
	Versus cirrhosis	Versus HCC	Versus chronicity	Versus HCC	Versus chronicity	Versus cirrhosis
**Epitope**	C5/C18		C11/NS3-3		C10/NS3-3	
**p.value**	0.030	0.011	0.045	0.043	0.018	0.047
**Epitope**	C5/E1-8		E2-3/NS4-1		C11/NS3-3	
**p.value**	0.041	0.017	0.017	0.013	0.007	0.043
**Epitope**	C5/NS3-3				C13/NS4-1	
**p.value**	0.030	0.009			0.026	0.000
**Epitope**	C5/NS3-6				C15/NS4-1	
**p.value**	0.009	0.013			0.029	0.047
**Epitope**	C6/C18				E1-3/NS4-1	
**p.value**	0.030	0.033			0.009	0.001
**Epitope**	C6/NS3-3				E2-4/NS4-1	
**p.value**	0.041	0.037			0.016	0.007
**Epitope**	C7/C18				E2-5/NS4-1	
**p.value**	0.019	0.046			0.029	0.002
**Epitope**	C11/C18				E2-11/NS4-1	
**p.value**	0.021	0.033			0.016	0.015
**Epitope**	C11/E1-8				E2-12/NS4-1	
**p.value**	0.021	0.041			0.003	0.022
**Epitope**	C11/NS3-3				NS2-2/NS4-1	
**p.value**	0.045	0.007			0.033	0.031
**Epitope**	E1-4/NS3-3				NS3-3/NS4-1	
**p.value**	0.019	0.041			0.020	0.007
**Epitope**	E2-12/NS4-7				NS4-1/NS4-3	
**p.value**	0.037	0.020			0.012	0.010
**Epitope**	NS4-2/NS4-7				NS4-1/NS5-4	
**p.value**	0.049	0.033			0.008	0.022
**Epitope**	NS4-3/NS5-1				NS4-1/NS5-7	
**p.value**	0.027	0.041			0.041	0.034
**Epitope**	NS4-7/NS5-4					
**p.value**	0.045	0.009				

^1^SPR ratio values are given in Additional file 3.

## Discussion

The aim of this study was to analyse the specific humoral response developed upon HCV infection, taking advantage of the potentialities of microarray-based screening systems. Previous studies dealing with anti-HCV Ab were informative but restricted to a global determination of their specificity because of the size of the probes that did not allow for precise identification of the epitopes. Moreover, the clinical parameters associated with the pathology that were taken into account were often restricted to the viral load, the response to the treatment or to two states of the disease, usually acute and chronic (Araujo et al. [2011]; Desombere et al. [2007]; Sillanpaa et al. [2009]). The use of peptide microarrays together with SPRi detection allows for more accurate identification of epitopes together with Ab quantification and is suitable for a large screening of both probes and serums (Cherif et al. [2006]). Three main reasons guided us to use 20aa peptides as probes to determine the specificity of anti-HCV Ab. First, peptide microarrays are more stable than protein arrays and are thus more suitable for large screening (Villiers et al. [2011]). Second, it is established that human Ab elicited by HCV infection are mainly directed against linear epitopes (Sallberg et al. [1992]). Third, it is usually considered that linear B cell epitopes typically vary from 6 to 20aa in length with a mean value of 13aa (Sollner [2006]).

The great immunogenicity of the Core protein (Figure 1), as revealed by using these peptides as probes, is in agreement with previous works using larger sequences (Araujo et al. [2011]; Chen et al. [1999]). This suggests that a large proportion of the Core epitopes are actually linear. This does not seem to be the case for the other viral proteins, especially envelope and NS2-NS3 proteins, since poor antibody responses against linear epitopes from these proteins are found in our study (Figure 1). Although we cannot exclude that this observation may partly result from the fact that the selected peptides do not cover the entire sequence of the protein (as opposed to Core-derived peptides), the low amount of Ab directed against these regions was also observed in other analyses using peptides as probes (Sallberg et al. [1993]). In contrast, authors using polypeptides or recombinant proteins detected anti-E1, anti-E2 (Chen et al. [1999]; Zibert et al. [1999]), and anti-NS3 (Hwang et al. [1996]; Yuki et al. [1994]) in more than 50% of the tested serums. Moreover, it is known that E1-E2 provide several conformational epitopes for neutralizing Ab (Giang et al. [2012]; Law et al. [2008]). In addition, and despite the fact that peptide sequences were chosen in regions exhibiting relatively conserved sequences across genotypes, the presence of three hypervariable regions in E2 makes it difficult to identify common epitopes. In the Core protein, the immunodominant region lies within the first 90aa, which are probably more exposed than the others as suggested by the hydrophobicity index (Gravy) of the sequence (-1.442 (aa 1–89) versus 0.313 (aa 90–189)). This is due to the presence of two regions with a high density of basic residues (leading to hydrophilic regions) in the N-ter portion of the protein (Klein et al. [2005]).

We first examined whether there was any correlation between the peptides recognized by serum Ab. Statistical studies by either PLS-DA or hierarchical clustering using Pearson’s correlation determine four groups of peptides (Figure 2). As expected, peptides that possess a 10-aa overlapping sequence tend to stand in the same group. However, the Core protein presents two independent regions with regards to its antigenicity, as previously observed (Nasoff et al. [1991]), which distinguish the first 80 aa (N-ter) from the rest of the molecule. This suggests a difference in the structure and/or the behaviour of the N-ter compared to the C-ter regions of the Core protein, which is supported by the difference in the hydrophobicity index between them. Analysis of aa sequences of different HCV genotypes shows three domains in the Core protein, one of them (N-terminal region) spanning residues 1–118 that roughly corresponds to the most immunogenic portion of the protein (Hope and McLauchlan [2000]). The observed correlation between the amounts of Ab against E1 and the C-ter part of the Core can be related to the correlation between circulating core and E1 levels (El Awady et al. [2006]).

Then we looked for a relationship between Ab specificity or amount and disease state. The very good separation between HCV- and HCV + patients obtained using hierarchical clustering (Figure 3) and PLS-DA (95% correct classification, Table 1) validates the methodology used in our study, especially the normalization of the signals. However, this approach is not sufficient for discrimination between each state of the disease, which would provide a good prognosis tool. Despite different Ab profiles in the serums from HCC versus chronic + cirrhotic patients, Ab profiles fail in affording correct classification of all patient samples, even after merging different disease states.

However, the lack of a predictive model does not mean a lack of significant differences between Ab profiles with regard to the disease states. The Mann and Whitney test reveals that only 4 peptides out of the 56 tested are recognized to different extents by Ab from at least two categories of serums (Figure 5): serums from cirrhotic patients display a significant difference in Ab amount against C15 and NS3-6 compared to chronic patients and against NS4-1 compared to HCC patients. However only one peptide, NS3-3, is recognized differently by the Ab from the three disease states. Three of these peptides belong to group 2, whereas none of them belong to groups 1 and 4. It is noticeable that all these 4 peptides lead to a low average SPR signal, resulting in a low (C15 and NS4-1) or null (NS3-3 and NS3-6) percent positive patients when focusing on easily detectable antibodies (SPR signal threshold > 0.5, Figure 1). It is possible that the high immunogenicity of the peptides of group 1 dwarfs differences between disease states. On the other hand, too weak immunogenicity, as for peptides from the envelope, can lead to the same result. Overall, our data demonstrate a very weak specificity of the Ab response with regard to the disease state, which is unlikely to be due to the linear nature of the epitopes tested. Indeed, a study using chimpanzees and large polypeptides derived from Core and NS3/NS4 concludes that Ab patterns are similar in persistently infected animals and those that cleared viraemia (Hilfenhaus et al. [1992]); also, acute and chronic infections are not discriminated in a study based on the measurement of Ab reactivity of human serums against polypeptides from Core and non-structural proteins (Araujo et al. [2011]).

We wondered whether other clinical parameters could account for the establishment of particular Ab profiles. Correlations with several parameters are highlighted by our study (Table 1). First, Ab patterns are related to Metavir A when scores 1 + 2 are merged, providing a predictive model allowing for more than 75% correct classification. The interdependence between Ab profiles and some clinical parameters is even stronger when considering ALAT values (correct classification > 90% - threshold = 2 N) and viral load (correct classification 100% - threshold = 600 000 UI). Few studies deal with the correlation between ALAT and anti-HCV Ab; Lohr et al. (Lohr et al. [1996]) observed that patients whose lymphocytes secreted anti-HCV *in vitro* had higher ALAT levels in serum, and Brillanti et al. (Brillanti et al. [1992]) observed a positive correlation between the ALAT and anti-HCV IgM levels. However, our result, in agreement with Nikolaeva et al. (Nikolaeva et al. [2002]), do not reveal any correlation between ALAT levels and anti-HCV titres. Further studies aimed at correlating anti-HCV Ab and viral load however they usually show a correlation between total anti-HCV Ab titres and viremic levels. This discrepancy between these studies and ours may be due to the probes used for anti-HCV quantification consisting of large recombinant proteins (Bassett et al. [1998]; Gane et al. [1999]; Yuki et al. [1996]) rather than peptides.

Thus, we demonstrate that although HCV infection elicits different Ab profiles in patients, only a very few linear epitopes are significantly different in the response they trigger by serums from chronic, cirrhotic, and HCC states. All these results led us to hypothesize that the different stages of the disease may be less correlated with the absolute value of the immune response against each viral protein than with the relative value of Ab recognition of different epitopes. This prompted us to analyse the ratio of SPR signals grouped in pairs of peptides (Table 2). This original approach improves the sensitivity of the analysis by reducing the impact of differences in the amplitude of humoral immune responses observed between patients. First, it reveals that Ab ratio for one peptide pair (C11/NS3-3) is an accurate marker of each state of the disease. Anti-NS3-3 alone was already identified in this study to separate the chronic state from a group consisting of cirrhotic and HCC states (Figure 5). Furthermore, association with anti-C11 allows for distinguishing cirrhotic from HCC states (Table 2). Second, a large number of peptide pairs discriminate chronic and HCC (15 and 14 pairs, respectively) from the two other states. Interestingly, we observed that the majority of the peptide pairs allowing for separation of the chronic state are intergroup (66.7%) In contrast, only 28.6% of the ratios used to discriminate between HCC and other states involve peptides belonging to different groups. In the first case, peptides from the Core protein are often implicated (66.6%) while NS4-1 is the most represented peptide in the second case (85.7%). Cirrhosis is the most difficult state to identify. This is not surprising since it is an intermediate state between chronicity and HCC.

Although direct profiling of Ab specificity does not provide an accurate prognostic tool, our study demonstrates that humoral response against HCV linear epitopes is modified according to disease state. This relationship does not result in a significant change in the overall immune response against individual epitopes. However, relative amplitudes of Ab responses against selected pairs of epitopes appear to be relevant in discriminating disease states. Overall, SPRi system for high-throughput screening has allowed for a thorough analysis of Ab specificity against multiple epitopes directly from patients serums. Our data reveal a set of peptides derived from HCV related to the state of the disease. This method opens potential further studies aiming to decipher the host- and pathogen-dependent molecular processes underlying the infections and the evolution of the resulting diseases.

## Electronic supplementary material

Additional file 1: **Clinical and biochemical characteristics of the HCV+ donors (infectant genotype 1b).** ND = Not determined. (PDF 52 KB)

Additional file 2: **Localisation of the selected peptides within the HCV sequence.** Peptide controls: C131 (from HCV) and HEL 83 (from hen egg lysosyme). (PDF 30 KB)

Additional file 3: **Peptide ratios allowing a separation of each disease state from the two others: SPRratio values (Data represent mean of three independent experiments).** (PDF 21 KB)

## Abbreviations

Ab: Antibody
ALAT: Alanine amino-transferase
HCC: Hepatocellularcarcinoma
HCC-OH: Alcoholic cirrhosis
HCV: Hepatitis C virus
PLS-DA: Partial least square determinant analysis
SPR: Surface plasmon resonance
SPRi: Surface plasmon resonance imaging.
