# Supplementary material for: Relationship between humoral response against hepatitis C virus and disease overcome
Source: Springerplus. 2014 Jan 27;3:56. doi: 10.1186/2193-1801-3-56 (PMC3915053; doi:10.1186/2193-1801-3-56)
Supplement: Supplementary file 1 — Additional file 1: Clinical and biochemical characteristics of the HCV+ donors (infectant genotype 1b). ND = Not determined. (PDF 52 KB) [file 40064_2013_796_MOESM1_ESM.pdf]

Additional file 1. Clinical and biochemical characteristics of the HCV+ donors (infectant genotype 1b). ND = Not determined

Chronicity

Patient	P1	P5	P9	P13	P17	P22	P26	P30	P36	P40	P44	P49	P54	P59	P64
Sexe	M	F	F	F	M	M	F	F	M	M	M	M	M	M	F
Age *	52	56	46	51	32	47	50	56	40	52	57	45	52	39	ND
Holding period (year)	28	26	22	30	22	30	26	28	23	18	22	25	34	21	ND
Metavir A	1	2	2	2	2	1	1	2	0	2	1	2	1	1	1
Metavir F	1	1	1	1	1	1	1	1	0	2	1	1	1	1	2
ALAT/ Norm ALAT	1.5	3.0	2.0	ND	13.4	0.8	1.4	1.5	1.1	1.4	0.9	1.3	1.3	1.3	ND
Viral load (Log UI)	6.3	4.4	5.3	7.1	6.4	6.1	5.7	6.6	5.8	ND	6.0	6.5	6.1	ND	ND

Cirrhosis

Patient	P2	P6	P10	P18	P23	P27	P31	P33	P37	P45	P50	P55	P60	P67	P71	P74
Sexe	M	M	M	M	F	F	F	M	M	M	F	F	M	M	F	F
Age *	47	46	48	51	69	54	59	53	55	ND	57	46	57	38	71	55
Holding period (year)	18	17	48	16	24	35	36	29	24	ND	23	21	36	36	46	15
Metavir A	3	1	3	3	3	3	1	2	2	2	2	2	1	2	3	1
Metavir F	4	4	4	4	4	3	4	3	3	4	3	3	3	4	3	4
ALAT/ Norm ALAT	1.8	3.1	3.5	3.7	2.6	ND	2.4	1.9	3.7	ND	4.1	1.3	3.7	5.3	3.9	0.7
Viral load (Log UI)	6.0	6.1	5.8	6.5	6.3	6.4	6.7	6.4	6.4	6.2	ND	5.8	5.3	5.0	5.9	6.8

HCC

Patient	P3	P7	P11	P11	P24	P28	P34	P46	P51	P56	P61	P63	P65	P72
Sexe	M	F	F	M	F	F	M	M	M	F	M	M	M	M
Age *	49	74	69	76	74	77	58	69	63	69	61	70	60	70
Holding period (year)	49	39	ND	ND	32	ND	45	ND	ND	48	29	44	ND	ND
Metavir A	ND	ND	2	3	2	1	1	2	ND	ND	2	ND	ND	ND
Metavir F	4	ND	4	2	4	4	2	4	ND	ND	4	ND	4	ND
ALAT/Norm ALAT	1.9	1.1	6.3	6.1	3.7	0.6	1.2	1.6	1.8	5.2	0.8	2.4	2.3	1.5
Viral load (Log UI)	5.6	6.1	7.1	6.5	ND	6.4	7.1	5.4	6.3	5.7	ND	6.8	ND	6.8

* : at the time of sample collection
