# Supplementary material for: Relationship between humoral response against hepatitis C virus and disease overcome
Source: Springerplus. 2014 Jan 27;3:56. doi: 10.1186/2193-1801-3-56 (PMC3915053; doi:10.1186/2193-1801-3-56)
Supplement: Supplementary file 2 — Additional file 2: Localisation of the selected peptides within the HCV sequence. Peptide controls: C131 (from HCV) and HEL 83 (from hen egg lysosyme). (PDF 30 KB) [file 40064_2013_796_MOESM2_ESM.pdf]

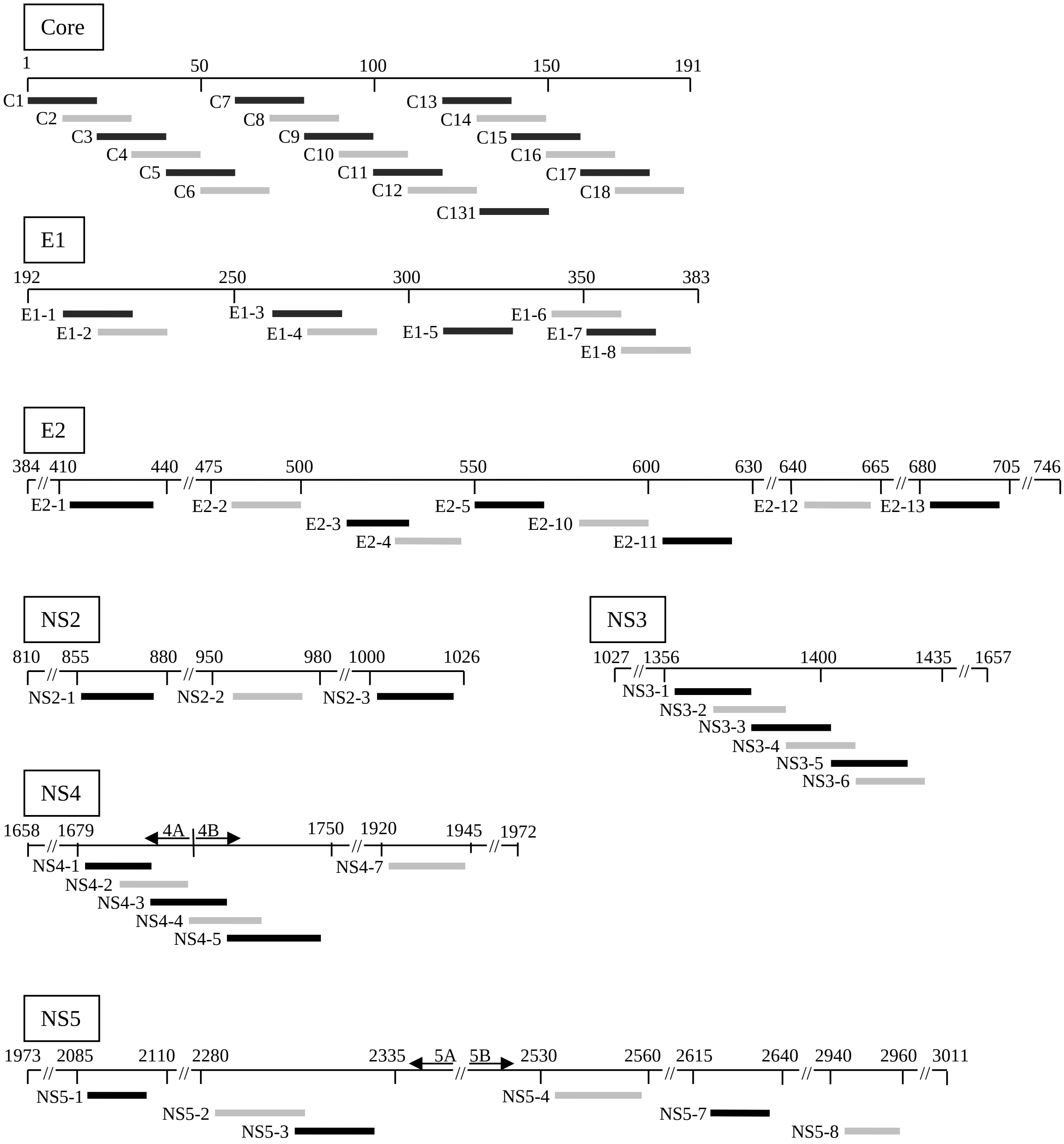


| | | | | | | | | | | | |
|---|---|---|---|---|---|---|---|---|---|---|---|
| C1 | 1-20 | C11 | 100-119 | E1-1 | 201-220 | E2-1 | 413-436 | NS2-1 | 856-876 | NS4-1 | 1681-1699 |
| C2 | 10-29 | C12 | 110-129 | E1-2 | 211-230 | E2-2 | 481-500 | NS2-2 | 956-975 | NS4-2 | 1691-1710 |
| C3 | 20-39 | C13 | 120-139 | E1-3 | 261-280 | E2-3 | 514-531 | NS2-3 | 1002-1023 | NS4-3 | 1700-1721 |
| C4 | 30-49 | C14 | 130-149 | E1-4 | 271-290 | E2-4 | 528-546 | NS3-1 | 1359-1380 | NS4-4 | 1711-1731 |
| C5 | 40-59 | C15 | 140-159 | E1-5 | 311-330 | E2-5 | 551-570 | NS3-2 | 1370-1390 | NS4-5 | 1722-1748 |
| C6 | 50-69 | C16 | 150-169 | E1-6 | 341-360 | E2-10 | 581-600 | NS3-3 | 1381-1403 | NS4-7 | 1922-1943 |
| C7 | 60-79 | C17 | 160-179 | E1-7 | 351-370 | E2-11 | 605-624 | NS3-4 | 1391-1410 | NS5-1 | 2088-2104 |
| C8 | 70-89 | C18 | 170-189 | E1-8 | 361-380 | E2-12 | 644-662 | NS3-5 | 1404-1425 | NS5-2 | 2284-2309 |
| C9 | 80-99 | | | | | E2-13 | 683-702 | NS3-6 | 1411-1430 | NS5-3 | 2307-2329 |
| C10 | 90-109 | C131 | 131-150 | | | | | | | NS5-4 | 2534-2558 |
| | | | | | | | | | | NS5-7 | 2620-2636 |
| | | | | | | | | | | NS5-8 | 2944-2959 |

**Additional file 2** Localisation of the selected peptides within the HCV sequence. Peptide controls: C131 (from HCV) and HEL 83 (from hen egg lysosyme)
