# Supplementary material for: Relationship between humoral response against hepatitis C virus and disease overcome
Source: Springerplus. 2014 Jan 27;3:56. doi: 10.1186/2193-1801-3-56 (PMC3915053; doi:10.1186/2193-1801-3-56)
Supplement: Supplementary file 3 — Additional file 3: Peptide ratios allowing a separation of each disease state from the two others: SPRratio values (Data represent mean of three independent experiments). (PDF 21 KB) [file 40064_2013_796_MOESM3_ESM.pdf]

Additional file 3. Peptide ratios allowing a separation of each disease state from the two others: SPRratio values (Data represent mean of three independent experiments)

	SPR ratios		
**Peptide ratios**	**Chronic hepatitis**	**Cirrhosis**	**HCC**
C5 / C18	6.45	3.50	3.00
C5 / E1-8	4.18	2.24	2.08
C5 / NS3-3	6.42	4.07	3.94
C5 / NS3-6	10.94	5.46	5.13
C6 / C18	8.09	7.16	5.64
C6 / NS3-3	8.70	9.73	5.20
C7 / C18	3.61	2.11	2.54
C10 / NS3-3	1.59	1.49	1.02
C11 / C18	3.21	1.79	1.71
C11 / E1-8	1.81	1.22	1.23
C11 / NS3-3	2.84	2.02	1.73
C13 / NS4-1	1.32	1.15	1.83
C15 / NS4-1	1.18	1.19	1.4
E1-3 / NS4-1	1.20	1.08	1.60
E1-4 / NS3-3	2.47	2.08	2.06
E2-3 / NS4-1	1.40	1.21	3.44
E2-4 / NS4-1	0.74	0.74	1.07
E2-5 / NS4-1	0.62	0.56	0?87
E2-11 / NS4-1	0.54	0.53	0.79
E2-12 / NS4-1	0.71	0.79	1.02
E2-12 / NS4-7	0.23	0.39	0.42
NS2-2/ NS4-1	0.75	0.77	1.08
NS3-3/ NS4-1	0.38	0.37	0.54
NS4-1/ NS4-3	0.90	0.88	0.65
NS4-1/ NS5-4	3.88	2.43	1.66
NS4-1/ NS5-7	0.92	0.95	0.71
NS4-2/ NS4-7	0.42	0.74	0.82
NS4-3/ NS5-1	0.77	0.89	0.88
NS4-7/ NS5-4	20.50	8.99	6.88
